# Non-Invasive Genetic Monitoring of Wild Central Chimpanzees

**DOI:** 10.1371/journal.pone.0014761

**Published:** 2011-03-15

**Authors:** Mimi Arandjelovic, Josephine Head, Luisa I. Rabanal, Grit Schubert, Elisabeth Mettke, Christophe Boesch, Martha M. Robbins, Linda Vigilant

**Affiliations:** Department of Primatology, Max Planck Institute for Evolutionary Anthropology, Leipzig, Germany; University of Uppsala, Sweden

## Abstract

**Background:**

An assessment of population size and structure is an important first step in devising conservation and management plans for endangered species. Many threatened animals are elusive, rare and live in habitats that prohibit directly counting individuals. For example, a well-founded estimate of the number of great apes currently living in the wild is lacking. Developing methods to obtain accurate population estimates for these species is a priority for their conservation management. Genotyping non-invasively collected faecal samples is an effective way of evaluating a species' population size without disruption, and can also reveal details concerning population structure.

**Methodology/Principal Findings:**

We opportunistically collected wild chimpanzee faecal samples for genetic capture-recapture analyses over a four-year period in a 132 km^2^ area of Loango National Park, Gabon. Of the 444 samples, 46% yielded sufficient quantities of DNA for genotyping analysis and the consequent identification of 121 individuals. Using genetic capture-recapture, we estimate that 283 chimpanzees (range: 208–316) inhabited the research area between February 2005 and July 2008. Since chimpanzee males are patrilocal and territorial, we genotyped samples from males using variable Y-chromosome microsatellite markers and could infer that seven chimpanzee groups are present in the area. Genetic information, in combination with field data, also suggested the occurrence of repeated cases of intergroup violence and a probable group extinction.

**Conclusions/Significance:**

The poor amplification success rate resulted in a limited number of recaptures and hence only moderate precision (38%, measured as the entire width of the 95% confidence interval), but this was still similar to the best results obtained using intensive nest count surveys of apes (40% to 63%). Genetic capture-recapture methods applied to apes can provide a considerable amount of novel information on chimpanzee population size and structure with minimal disturbance to the animals and represent a powerful complement to traditional field-based methods.

## Introduction

Obtaining reliable estimates of a species' population size is an important component in determining its conservation status and provides a baseline for evaluating demographic change and/or conservation success over time. Rare and elusive species living in low visibility environments, like many of the world's threatened tropical animals, are usually impossible to count directly and difficult or expensive to detect by indirect methods [1], [2]. It is clear that most African primates are in decline due to habitat destruction [3], [4], disease [5], [6] and the commercial bushmeat trade [7], [8], although the extent and magnitude of this decline is largely unknown [1], [9]-[11].

Great apes have been particularly difficult to survey due to their shy nature, low densities and occurrence in remote and inaccessible areas. Furthermore, ethical and practical concerns regarding trapping and collaring animals which are cognitively advanced, socially-bonded and susceptible to human disease has prevented the use of certain population estimation techniques, such as direct counts or capture-mark-recapture [1], [12]. To circumvent these difficulties, ape surveys are done by counting ape sleeping nests and/or dung piles along transects and transforming these data into estimates of abundance or density. However, due to variability in nest creation and decay rates, as well as some difficulty in distinguishing the nests of sympatric chimpanzees and gorillas, conversion of indirect ape signs into ape numbers can yield accurate, but imprecise, estimates (i.e. the true population size falls within the bounds of the minimum and maximum estimate, but the width of this range of values is large) [13]-[18]. Estimation of site-specific nest construction and decay rates as well as information on nest location and forest type for discriminate function analysis can improve the precision of traditional ape surveys [14], [15], [17] but collecting these additional data requires months of work by well-trained field researchers. The resolution achieved is sufficient for detecting catastrophic ape declines [3], [7] but in order to detect more subtle changes, improvements in ape monitoring methods are required [1], [14], [16].

Faeces, hair, feathers and other non-invasively collected materials are reliable sources of DNA and have allowed evolutionary and ecological processes to be inferred for elusive species [19], [20]. Genetic-based approaches require additional laboratory expense, time and expertise compared to traditional field-based methods, thus the amount of information derived should be proportionately beneficial to the increased expense. By generating individual-specific genotypes, non-invasive genetic studies have evaluated the effective population size of species, inferred their dispersal patterns and assessed their genetic diversity and thus provide a powerful biomonitoring tool for populations with minimal perturbation to the species under study [19]-[21]. A comparison of genetic and standard indirect methods for population estimation of various species shows that both over and under estimation of the true population size occurs with the latter [22]-[25] and that the genetic method can yield more precise results as well as information on group membership and movements [26]. Furthermore, studies evaluating genetic capture-recapture estimators using simulated data or direct counts of individuals have found them to have a high degree of precision and accuracy in most situations [27]-[30].

Currently, the vast majority of our knowledge on the behavior and ecology of chimpanzees comes from long-term studies on the eastern and western subspecies (*Pan troglodytes schweinfurthii* and *P.t. verus*, respectively) [31]. Very little is known about central chimpanzees (*P.t. troglodytes*), as continuous, long-term habituation and study began only recently [31]-[34]. All chimpanzee populations appear to share some basic characteristics including male philopatry, fission-fusion social grouping and territoriality, with males actively defending their group's territory through boundary patrols and by making incursions into adjacent territories and aggressing neighbors [31], [35], [36]. Without habituation we would know virtually nothing about the life history patterns and behavioral ecology of wild chimpanzees. However, it requires years of intensive work and is generally accomplished for only one or a few chimpanzee groups in any area. Furthermore, although the presence of researchers has been shown to have a positive impact on the conservation of apes [6], [34], [37], [38], the possibility of lethal disease transmission from human observers to apes has become an increasing concern at several sites [6], [12]. Thus, ways of maintaining a research presence with minimal disruption is a desirable goal of future research initiatives so that multiple, adjacent ape communities can be studied without habituating all groups under investigation.

Non-invasive genetic sampling of apes offers a complement to traditional field-based approaches for understanding some aspects of wild chimpanzee society. Studies on kin relationships and patterns of relatedness within and between social groups [39]-[44], relative levels of genetic diversity [45], [46], and community composition [32],[47] have all been undertaken using non-invasive sampling on habituated and unhabituated eastern and western chimpanzees. These studies feature very limited sampling of adjacent groups because only a single or few habituated groups are studied, or because group membership is unknown due to the fission fusion social system of the species. Repeated genetic sampling over space and time can be used to estimate group sizes of multiple unhabituated ape groups over a larger area, thus allowing for a better understanding of their population dynamics [26].

In this study, we aimed to estimate the number of chimpanzees and their distribution into groups in a 132 km^2^ area of Loango National Park, Gabon using the genetic capture-recapture method. To do so, we amplified 8 rapidly evolving, highly variable, autosomal microsatellite markers from central chimpanzee faecal samples collected opportunistically over a four-year period, which allowed us to reliably distinguish even closely related individuals. Because chimpanzees are male-philopatric with males remaining in their natal community for life, we also amplified 13 Y-chromosome microsatellite markers for all males. We hypothesized that the resulting paternally inherited haplotypes should be the same or similar within groups, while differing between groups, as has been previously observed in a study of multiple communities of eastern chimpanzees [45]. Using the autosomal genotypes from all individuals and Y-chromosome haplotypes from identified males, we determined the number of chimpanzee communities in the area, minimum group membership, and minimum territory size, and along with data from the field, identified repeated cases of intergroup violence.

## Materials and Methods

### Study site and sample collection

Samples were collected across the Loango Ape Project research site, a 132 km^2^ area in the central sector of Loango National Park, Gabon [32]. The study area contains sympatrically-living central chimpanzees and western gorillas (*Gorilla gorilla gorilla*) and is part of the westernmost distribution of both sub-species.

Between February 2005 and July 2008, two to four field teams conducting ape habituation and biomonitoring activities in the study area opportunistically and unsystematically collected up to three-day-old chimpanzee faecal samples; due to the presence of dung beetles, rain and maggots, ape faeces do not persist for more than three days at Loango. Faeces were preserved using the two-step ethanol-silica procedure [48]. The geographic coordinates of each faecal sample were recorded using a Garmin GPSMap® 60 or 60CSx.

A total of 452 putative chimpanzee samples were collected from beneath night nests and from where chimpanzees had defecated as they moved through the forest during the day [26]. As previously described in detail [26], we included our putative chimpanzee genotypes and 13 genetically identified gorilla genotypes from the study site in a STRUCTURE 2.1 Bayesian model-based clustering program analysis [49] to confirm that samples were of chimpanzee origin and not misidentified gorilla faecal remains. These analyses revealed that a small proportion (5%) of chimpanzee faecal samples were misidentified in the field as being of gorilla origin and (2%) vice versa, resulting in a total of 444 collected chimpanzee samples.

### DNA extraction, quantification and amplification

Faecal samples were extracted from one month to one year after collection, using the QIAmp Stool kit (QIAGEN) with slight modifications [48]. DNA quantification was performed as described in [50]. To determine the sex of the individuals, Three to four independent amplifications from each DNA extract were performed for a segment of the X-Y homologous amelogenin locus in a one-step polymerase chain reaction (PCR) which allows for sex identification of the samples [51]. Extracts that failed to amplify at the amelogenin locus were not analyzed further. For all other extracts, at least three independent amplifications from each DNA extract were performed at 8 microsatellite loci (Table S1, [26]) along with a minimum of five negative controls, using a two-step multiplex PCR method described in detail elsewhere [52]. Extracts that produced genotypes at three or fewer loci after the first set of PCRs were no longer used. Some low-quality extracts which yielded confirmed alleles at four or five loci after six independent PCR amplifications were run in quadruplicate in a 60μl two-step multiplex PCR as described in [26]. In a few cases, extracts still amplified poorly and only one of the two alleles could be confirmed for some loci, making it impossible to assess whether the samples originated from one, or multiple individuals. Assuming that these loci amplified poorly because of locus-specific DNA degradation, these extracts were genotyped at 3 additional autosomal microsatellite loci known to amplify in chimpanzees (D1s1622, D1s1656, D4s1627,[52]) with the intent of obtaining more genotypic information for the samples.

At least one sample from each male individual identified in the data set was further genotyped at the 13 Y-chromosome loci previously described in [32] (Table S2) using a two-step multiplex PCR method detailed in [52]. Nested reverse primers were designed for the Y chromosome loci for use in the second step of the multiplex PCR, as nesting primers is theorized to improve multiplex amplification success ([53], Table S3).

Up to four different PCR products were combined and electrophoresed on an ABI PRISM 3100 Genetic Analyser and alleles were sized relative to an internal size standard (ROX labeled HD400) using GeneMapper Software version 3.7 (Applied Biosystems). Heterozygous genotypes were validated by observing each allele in two or more independent reactions and depending on the quantity of DNA in the extract, homozygous genotypes were confirmed in up to five independent PCR amplifications [52]. Furthermore, Y-chromosome alleles were corroborated in at least two independent PCRs. To visualize the genetic distances and relationships between the Y-chromosome haplotypes, Network 3.0 (www.fluxus-engineering.com) was used to construct a median joining haplotype network with all loci equally weighted.

### Discrimination of individuals

We used CERVUS 3.0 to identify independent samples with matching autosomal genotypes. We estimated the minimum number of autosomal loci necessary to obtain a P_IDsibs_ value of ≤0.001 [54] and thereby attain high confidence that any two matching samples originated from the same individual and not from full-siblings. Matching genotypes were then given a consensus ID (“C” followed by a number) and composite genotype for use in subsequent analyses. Genotypes from different samples mismatching at three or fewer loci were re-examined for possible genotyping errors and in some cases additional genotyping was undertaken to resolve any ambiguities.

### Chimpanzee group composition and minimum territory size

**Figure 1:**
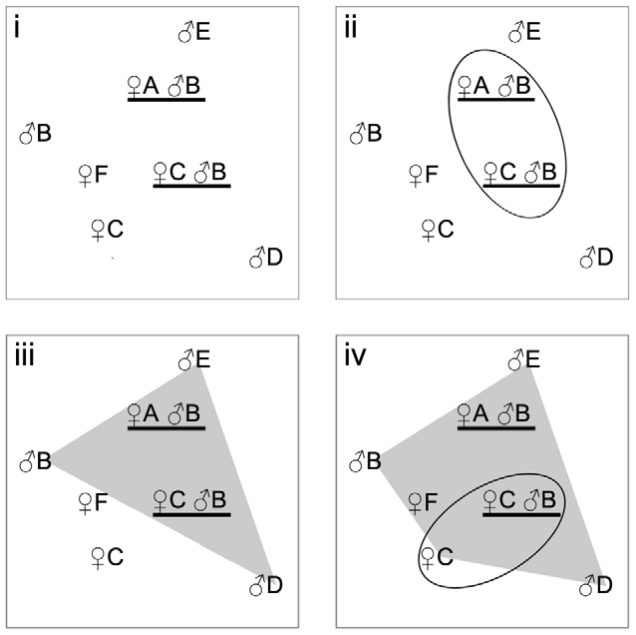
Determination of chimpanzee group composition and minimum territory size. Each letter represents an individual's genotype and its sampling location over the course of the entire study period. Females are denoted by ♀, males are denoted by ♂. Underlined samples were found at the same location on the same day. (i) Relative geographic locations of samples from individuals A through F (ii) Assuming that individuals found together belong to the same group, here male B links together samples A and C, thus A,B & C are all members of a single community. (iii) If males B, E & D all carry the same Y-haplotype we assume they belong to the same community and draw a minimum convex polygon (MCP) around these individuals. As chimpanzees are territorial, we assume that females found within this MCP belong to the males' community. Thus, individuals, A, B, C, D & E all belong to the same community. (iv) Because female C was found within the Y-haplotype defined MCP at one collection event, we can extend the MCP to include any other sampling events of female C. By doing so, female F now also falls within the MCP of the group so that individuals A, B, C, D, E & F all belong to the same group. See text for exceptions to these rules.

The number, composition and minimum territory size of chimpanzee groups were determined using the following criteria (illustrated in Figure 1). First, as in [26], samples from individuals collected on the same day at the same GPS location (same nest site or multiple fresh faecal remains found together) were considered to belong to individuals from the same group.

Second, as chimpanzees are a patrilocal species we hypothesized that if males of each community carry a unique set of Y-chromosome haplotypes as suggested by previous research [45], then these groups of Y-chromosome haplotypes would cluster together according to chimpanzee territories. We coded all male samples by their Y-haplotypes and plotted the sample collection locations onto a map of the study area using ESRI® ArcMap™ 9.2. We then drew minimum convex polygons (MCPs) around each unique set of haplotype clusters using the MCP tool implemented in the Hawths Analysis Tools v. 3.26 software package.

Third, females and males found within a given Y-chromosome-delineated MCP community were attributed to that community, as we assumed no territory overlap. Individuals found in association with samples from different group affiliations over the study period could not be attributed to any community and were not used in MCP construction. When a female was found both within and outside of an MCP, the MCP was redrawn to include the exterior female data points. The area covered by the final MCP was considered the minimum territory size for that chimpanzee community.

If females did not fall into any MCP and were either found alone, with other females who were also not attributed to any group, or from a collection site where only the one sample contained usable DNA, then they could not be attributed to any group. Because a dead individual has ‘left’ the population this violates the assumption of closure in our population and we do not include individual C12 (known to have been killed in an intercommunity attack in August 2005 [32]) in the mark recapture calculation. The individual is however relevant to the investigation of group dynamics and is included in the determination of group membership.

### Chimpanzee genetic capture-recapture population estimation

Grouping all samples into a single-sampling session scheme and using individual genotypes that were identified from one (initial capture) or more (recaptured) samples, we calculated a genetic capture-recapture estimates using the maximum likelihood two innate rates model (ML-TIRM) estimator implemented in the software Capwire (www.cnr.uidaho.edu/lecg) [27]. The approach assumes a closed population and a recapture probability equaling the capture probability but also accounts for capture heterogeneity as it divides individuals into those with high or low capture probabilities [27]. Capwire calculates 95% CIs using the parametric bootstrap [27]. In a previous study where gorilla faecal samples were collected with the same methods as in this work, it was found that due to heterogeneity in the opportunistic sampling protocol the ML-TIRM estimator is the most conservative of the available published estimators [26], while the other methods (rarefaction curve [55], sequential Bayesian estimator [28] and ML-Even Capture Model implemented in Capwire [27]) appear to underestimate the population size ([26] and unpublished data).

Calculating a population estimate using samples collected over the entire four-year study period may violate the assumption of closure in our models. Thus, to compare inferences made over the entire study period with those from a more restricted time period (and consequently smaller spatial area), we calculated a population estimate using samples collected from February 2005 to June 2008 as well as a population estimate from samples collected from each year separately.

## Results

### Discrimination of individuals

In total 202 chimpanzee samples yielded usable genotypes, resulting in a 46% (202/444) extraction success rate over the four year period. Extraction success was not obviously related to time of year (data not shown) and was consistently low every year, ranging from 41% (in 2007) to 63% (in 2005).

Genotypes from the 202 samples were on average 98.9% complete with 88.6% of extracts (179/202) genotyped at all eight loci and 9.9% genotyped at seven loci. After identifying matching genotypes from multiple samples and assigning consensus names to the matches, genotypes from the resulting 121 chimpanzees were on average 99.5% complete (Table S1).

In all cases where two or more samples produced identical genotypes at seven or all eight loci, we obtained a PIDsibs value of ≤0.001, strongly suggesting that in these cases the samples did indeed come from the same individual and were not derived from full-siblings who happened to be identical at these loci. Two samples C74 and C120 produced confirmed genotypes at 6 of the 8 loci with only 1 allele confirmed at the other two loci. However, both of these samples mismatched all other samples at a minimum of 4 loci so we are quite certain that they represent unique individuals.

The rates of allelic dropout and the appearance of irreproducible, sporadic alleles were calculated and found to be on average 16% and 2% per PCR, respectively. Using the multiple tubes approach with DNA quantification we estimated the number of independent PCRs necessary to ensure with >99% certainty that homozygote genotypes are authentic and not the result of allelic dropout [50], [52]. We found that 4, 3 and 2 independent PCRs for extracts containing 1–10 pg/µl, 11–25 pg/µl and more than 26 pg/µl DNA concentrations, respectively were required. Furthermore, we examined the mismatch distributions for the complete set of genotypes (up to 11 autosomal loci and the Y-haplotype, Figure S1) and found that no individuals mismatched at only one locus and only two pairs of individuals mismatched at two loci and these were confirmed through PCR replication as recommended [20], [56]-[58]. Furthermore, in cases where multiple samples from the same male were genotyped at the Y-chromosome loci, the resulting haplotypes were always identical for any given male, further indicating a low-level of genotyping error. We are thus confident that the number of single captures we obtained in the study reflect the actual number of individuals present in the population and are not an artifact of genotyping error.

### Chimpanzee Y-chromosome haplotypes

Six of the 13 Y-chromosome microsatellite loci under investigation were polymorphic, although only two alleles were seen at each of these six loci (Table S2). After combining haplotypes generated using different samples that proved to originate from the same individual, individual haplotypes were on average 95.1% complete. Nine haplotypes were observed in total and labeled from A to I. Each haplotype was observed from at least two samples except haplotype I, which was observed in only one sample (C134). Because of the unique allele this sample exhibited at locus Dys510, six successful PCRs were used to confirm the haplotype. Due to the low amount of variation detected with the Y-chromosome loci, haplotypes often differ by only one mutation and five mutations at most (Figure S2).

### Chimpanzee group composition and minimum territory size

**Figure 2:**
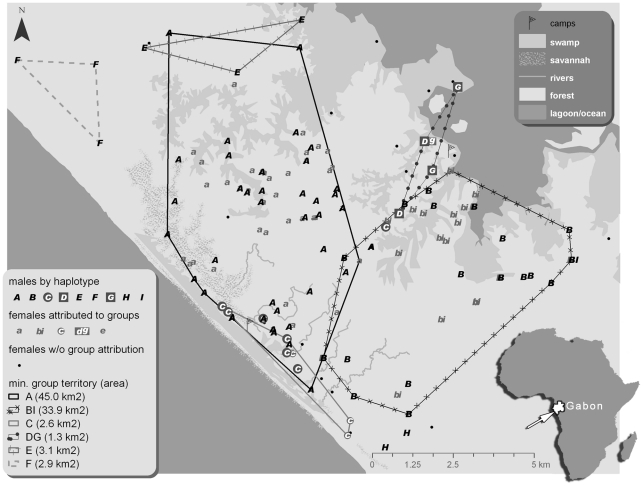
Map of Loango research site, geographic location of all faecal samples in study and the seven Loango chimpanzee groups. Males are designated by their Y-chromosome haplotype (uppercase A-I). Females are designated by the lowercase letter(s) of the group in whose minimum convex polygon (MCP) they were found (a, bi, c, dg, e, f or h). Females that did not occur in any MCP or that were found in association with more than one group throughout the study are represented by black circles. In cases where females were found both within and outside of a given MCP, the MCP was enlarged to include the “exterior” geographic location of the female. MCPs represent minimum territory boundaries of each chimpanzee community. Area of MCPs stated in parentheses in legend. For group H (southern most points) only 2 individuals were identified and so no MCP could be drawn. Inset, map of Africa with Gabon highlighted in white, arrow indicates location of Loango field site.

Groups were first identified by visually evaluating whether Y-chromosome haplotypes of the 58 males (N = 92 observations) in the study area clustered geographically. Males carrying haplotypes E, F and H appeared to each cluster geographically to the exclusion of all other haplotypes (Figure 2). MCPs were drawn using the geographic location of the males from each haplotype group (groups are named according to the Y-haplotype(s) that define them). As only two males, sampled once each, carried the H haplotype no MCP was obtained.

**Figure 3:**
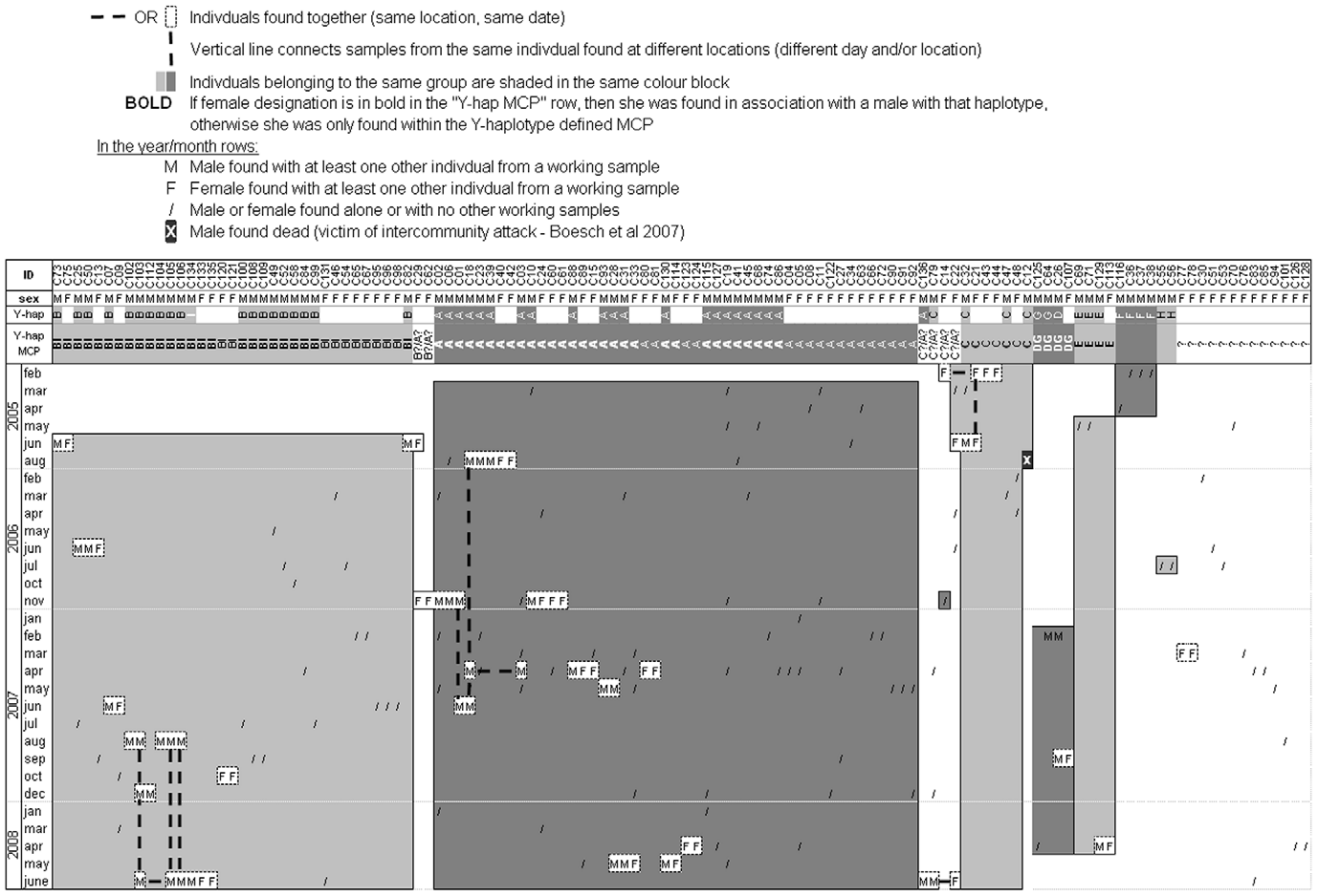
Loango chimpanzee groups and composition over the 4-year study period. ID is consensus name given to matching chimpanzee genotypes. In row “sex”, M = male, F = female. Y-hap refers to the Y-chromosome haplotype of the male individual. Y-hap MCP is the group membership identity of each individual based on their exclusive inclusion in any of the Y-haplotype defined MCPs. B?/A? means individual may belong or have belonged to either group B or A over the study period. A?/C? individual may belong or have belonged to either group C or A. “?” indicates females could not be attributed to any group. Grey boxes bound first and last instance when group members detected over the 4-year study period.

The 20 males carrying haplotype B also clustered to the exclusion of all other male haplotypes except in one instance where the male with haplotype I (C134) was found with three males with haplotype B (C103, C105, C106) (Figure 3). We therefore consider group BI to include all males with haplotype B and the male with haplotype I, as well as all the females that fall within the group BI MCP.

Similarly, haplotypes D and G co-occur within a very small geographical space (Figure 2), although with only two sampling locations per haplotype it is not possible to observe clustering of the two haplotypes and it is parsimonious to assume that these two haplotypes belong to a single group, DG.

As shown in Figure 2, most of the group C MCP occurs within the southeastern portion of the group A MCP. This overlap is primarily driven by male C45 (southeastern-most point of MCP A). We consider groups A and C separate, as we assume that groups containing more than one haplotype should not show geographic differentiation of the two haplotypes. In other words, if males with the A and C haplotypes belonged to the same group, we would expect the C haplotypes to be present in more than just the small southeastern portion of the A territory.

The 47 females falling within these MCPs were considered to belong to their respective Y-haplotype defined groups. Four females (C14, C22, C29 and C62, see below) had ambiguous group affiliations and 13 females (listed at the end of Figure 3) were all found outside MCPs and could not be attributed to any group.

Behavioral and genetic evidence also suggest that groups A and C are distinct entities. In addition to the purported August 2005 killing of C-haplotype male C12 by group A males reported in [32], other observations suggest intercommunity violence between groups A and C. In June 2006 and June 2007 there were two probable infanticides in the group A and C overlap zones (Figure S3). The genetic tracking evidence also suggests interactions among members of groups A and C. Females C14 and C22 were first found in association with group C individuals and later on within the group A MCP (Figure 3, Figure S3). Finally, haplotype C male C79 was initially found in the center of the haplotype C MCP then in the northeastern limit of group BI's MCP and then, as described above with a haplotype A male (C136) (Figure S3). Chimpanzee males have rarely been observed to transfer between groups even in the case of group dissolution [36], [59], [60], making the tracking of this male highly intriguing. Similarly, female C29 was found first with a B haplotype male and then later on with three C haplotype males and another female (C62) (Figure 3). Thus, C29 and C62 were not attributed to any group (Figure 3), the behavioral data from the collection site also suggests a possible intercommunity encounter between groups BI and C as sprayed diarrhea and several broken and partially uprooted samplings were found on site. Samples collected on these dates were not included in MCP construction.

In sum, seven groups were identified (A, BI, C, DG, E, F and H), however only groups A, BI and C were detected on more than 10 occasions (89, 47 and 11 times, respectively), making inferences about minimum group size and territory size limited to these groups. Thus, minimum group size ranged from 7 to 47 individuals, and minimum territory size ranged from 2.6 km^2^ to 45.0 km^2^ (Table 1).

**Table 1:** Summary of minimal inferred group composition and minimum territory size.

Group	Minimum # individuals	Minimum # males	Minimum # females	Minimum territory size	# occasions group detected
**A**	47	21	26	45.0 km^2^	89
**BI**	35	20	15	33.9 km^2^	47
**C**	7*	3*	4	2.6 km^2^	11*
**DG**	4	3	1	n.d,	5
**E**	4	3	1	n.d.	4
**F**	4	4	0	n.d,	4
**H**	2	2	0	-	2
**ungrouped females**	17	-	17	-	24
**ungrouped males**	2	2	-	-	4
**Total**	122*	58*	64	-	190*

Group H was only detected twice, so no minimum territory size could be calculated. n.d. – not determined as groups were sampled fewer than 10 times.

*- an additional dead male (C12, from group C) was identified in a previous study and included in the totals presented.

### Chimpanzee genetic capture-recapture population estimation

**Figure 4:**
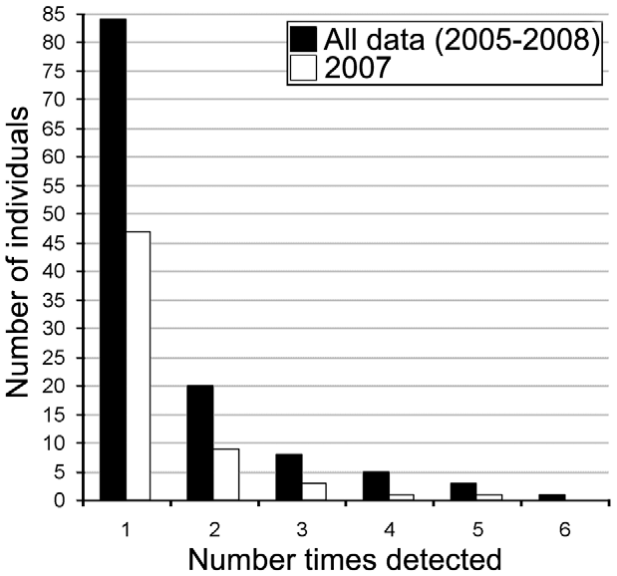
Frequency of detection of individual chimpanzee genotypes during the study period.

Of the 202 chimpanzee samples from the study site yielding usable genotypes, there were 13 instances of samples collected at the same location and on the same day as the other samples representing the same individuals; these cases were collapsed into single captures. Of the 83 samples collected in 2007, 61 unique genotypes were identified. The number of faeces successfully genotyped per individual ranged from 1 to 6 (mean 1.56, SD 1.05) for the entire study period and from 1 to 5 (mean 1.36, SD 0.80) for the 2007 sampling period, with the majority of individuals sampled only once in either sampling schemes (entire study period: 84/121; 2007 sampling period: 47/61) (Figure 4). In 2005, 2006 and 2008 only 39, 35 and 32 usable samples were collected respectively with very few recaptures obtained (15, 8 and 2 respectively), making a reasonable and biologically relevant population estimate from these sampling years unfeasible.

Applying the ML-TIRM population estimator, resulted in a point estimate of 283 (CI_TIRM-AllData_: 208–316) chimpanzees using the 132 km^2^ area over the entire study period and 176 (CI_TIRM-2007_: 113–220) chimpanzees using a 73 km^2^ subset of the study area in 2007 alone. By adjusting for area sampled, we obtain similar density estimates from the entire data set and the 2007 data: 2.14 (CI_TIRM-AllData_ 1.58–2.39) chimpanzees/km^2^ and 2.41 (CI_TIRM-2007_ 1.55–3.01) chimpanzees/km^2^, respectively. The precision of the estimates, measured as the entire width of the 95% confidence interval divided by the estimate itself, was 38% and 61% of the point estimate for the entire data set and 2007 samples, respectively.

## Discussion

### Y-chromosome haplotypes

Of the 13 Y-chromosome loci genotyped, only six were variable, and then, only dimorphic. This low amount of Y-chromosome variation differs from the pattern observed in eastern chimpanzees in Kibale forest, Uganda [45] and western chimpanzees in Tai National Park, Cote d'Ivoire (G. Schubert, personal communication) when the same set of genetic markers were used. As the Y-microsatellite loci were originally developed in humans [61] and further refined in bonobos [62], it is unlikely that these markers are more variable in eastern and western chimpanzees due to ascertainment bias. The low variability could be due however to various, non-mutually exclusive reasons. First, a small number of chimpanzees may have colonized the area in the recent evolutionary past and subsequently fissioned into the various groups present today. In this scenario, mutational processes have simply not had enough time to generate the high amount of variation observed in other chimpanzee populations. A recent colonization is feasible considering the relative remoteness of the research area, bordered in the west by the Atlantic Ocean and the east by a large lagoon. Had poaching or disease extinguished the past chimpanzee groups in the area, or if the habitat only recently became suitable for chimpanzees due to expansion of forest refugia [63], a recent colonization of the area is a reasonable possibility. Second, if male reproductive skew is much higher than that previously reported for eastern [40], [41], [64], [65] and western chimpanzees [39], [43], it is possible that paternally-related chimpanzee male lineages can dominate reproduction and effectively decrease the amount of Y-chromosome variation in the population. Finally, a population bottleneck, past selective sweep across all or part of the central chimpanzee Y-chromosome or other evolutionary pressure not acting in eastern or western chimpanzees could also explain the low diversity of the Y-chromosome haplotypes observed at Loango.

### Chimpanzee group composition and minimum territory size

As compared to other chimpanzee subspecies, very little is known about social organization and grouping patterns in central chimpanzees [31], [34], [36]. Using autosomal and Y-chromosome genetic data from non-invasive samples collected opportunistically over four years, we show that information regarding group number, minimum composition and territory size can be obtained without direct observation. We were able to identify seven groups in the study area by using the geographic clustering of Y-chromosome haplotypes, which we hypothesized would occur if haplotypes were not shared between groups as documented in eastern chimpanzees [45]. Four of the groups (A, BI, C and DG) appear to utilize the majority of the research area whereas the territories of groups E and F probably extend beyond the northwestern limits of the research area and group H's territory extends southeastward. Approximately 38% of females could not be attributed to any group however, emphasizing the need for extensive sampling in studies of this kind in order to obtain accurate group membership information.

Several considerations suggest that the MCPs derived here may underestimate the territory sizes of the chimpanzee groups at Loango. MCPs can overestimate the territory size of species by including areas that are not used by the individuals [66]. On the other hand, in our study, 54% of samples did not contain sufficient amounts of DNA for genetic analysis so some samples falling outside the obtained MCPs could not be used. Additionally, we assumed and observed little territory overlap, although generally it is reported to be 7.5% or more [67]. It appears that for the two groups for which we have the most data, A (45.0 km^2^) and BI (33.9 km^2^), the territories are within the range of known chimpanzee territory sizes (13–50 km^2^ in western chimpanzees and 4–38.3 km^2^ in eastern chimpanzees, [31]. It is possible that haplotypes A and/or B are present in two adjacent groups, so that neither haplotype delineates a single group but two parts of a recent group fission [68]. Arguing against this possibility is the occurrence of males C18, C19 and C23 in the northern/middle part of the territory and in the coastal/southern part of the A group MCP over the course of the study, thus suggesting they are using the majority of the A territory and are not restricted to any one part of it (Figure S4). Using samples only from males with the A haplotype that were sampled more than once to construct the A MCP, we still obtain a moderate territory size of 24.4 km^2^ (Figure S4). The four samples collected in the northern part of the A territory were only sampled once so confirming their membership in, and the entire territory size of, group A remains more tenuous. Individuals from group BI were rarely recaptured so no samples were captured in both the southern and northern part of the BI MCP. Chimpanzees living in savanna-woodland or savanna-riverine forest habitats tend to have larger territories than true forest dwelling chimpanzees [31]. Loango contains heterogeneous habitat which may partially explain why the territories may be as large as some of the bigger ones observed in other chimpanzee populations.

Most of group C's small territory is overlapped by group A's territory. The circumstantial evidence suggests that group A is expanding its territory and replacing group C by making incursions into group C's territory and killing group C males and infants. A similar pattern of group extinction was observed in two eastern chimpanzee populations. At Mahale, one group (M) was suspected of exterminating the males of another (K), co-opting many of the group females and expanding into its territory over a 12 year period [59], [69]. Similarly, at Gombe, after the fissioning of the main study group into two distinct entities, the Kasakela group exterminated the males of Kahama group, expanded into their territory over the course of four years and acquired at least one female from the exterminated group [68], [70] **.** Additionally, at a third eastern chimpanzee field site, the Ngogo study group has been killing neighboring individuals over the past 10 years and subsequently expanding into their territory [71].

As an alternative explanation for the apparent overlap between groups A and C, it has been suggested that neighborhoods exist in some chimpanzee communities [72], [73], and so it is theoretically possible that males with haplotype C constitute a small neighborhood within a community that includes males with haplotypes A and C. Intragroup infanticides and violence have been reported for chimpanzees in other populations [74], [75], making this scenario possible. However, the distribution of Y-chromosome haplotypes in chimpanzee communities known to exhibit male neighborhoods has not yet been investigated so it is unclear whether a geographical clustering of haplotypes, as observed here, would be expected. The location of aggressive encounters in the zones of territory overlap (Figure S3), is also indicative of inter-, rather than intra-, group dynamics [36], [76]. This suggests that groups A and C are indeed distinct, with group C males and infants possibly being exterminated by group A individuals, and group C females moving to group A (i.e. females C14 and C22) or other groups (i.e. females C21, C43, C44 and C48 were never recaptured after May 2006) and group A expanding into the group C's territory. This pattern is highly similar as to what was observed in the K group extinction at Mahale [59], [69] and suggests that such intense intergroup aggression is also part of the central chimpanzee's behavioral repertoire [32], [36].

### Chimpanzee population estimate by genetic analysis

We show in this study that despite reliance on opportunistically collected faecal samples with poor extraction success (46% on average), we can obtain useful population estimates, albeit with moderate 95% confidence intervals. As gorilla samples collected in the same manner from the same site over a similar time period had a higher success rate (82%, [26]), and chimpanzee samples from other research sites have similarly high success rates [43], [48], we suspect that some component of the Loango chimpanzee diet reduces preservation and/or inhibits amplification of chimpanzee DNA [77], [78].

Using the ML-TIRM method we obtain a population estimate of 283 chimpanzees (ranging from 208 to 316 individuals) using the Loango study area from 2005–2008. Chimpanzees are long-lived primates with slow life histories. Adult deaths and female dispersals are rare events and chimpanzee females give birth only once every 5 to 6 years [31]. Furthermore, infants (0–5 years) are likely absent in our sample as faecal samples from this age class are notoriously difficult to obtain even for habituated chimpanzees. Thus it does not appear that using all 4 years of data grossly violates the assumptions of closure inherent in the population estimation model since the estimates from the entire data set and from just the 2007 samples were similar when correcting for area sampled. Similarly, in an analysis of the sympatric western gorilla population at Loango, we previously showed that using a three-year dataset gave a similar population estimate as when using only a given 12 month period [26]. Knowing that samples collected in successive years can be combined to obtain a population estimate is encouraging, as no estimate could be calculated for three of the four study years because an insufficient number of samples were available due to the poor extraction success rate. We can state with confidence that at least 122 chimpanzees (including the dead male identified in [32]) used the research site from 2005–2008 as this was the number of unique genotypes identified in the area.

The density estimate of 2.14 chimpanzees/km^2^ (range: 1.58–2.39) is in the upper range of those previously reported for other central chimpanzee sites [0.03–2.78 chimpanzees/km^2^, 14]. It is important to note however that published chimpanzee density estimates are from nest surveys which have been shown to underestimate the density of chimpanzees by 70% or more when compared to estimates obtained from direct observations in eastern and central [14] but not western chimpanzees [17], [79]. This implies that the true densities of chimpanzees may be higher than currently estimated with traditional methods.

Most individuals were only captured a single time, resulting in a population estimate with moderate precision. Consequently, the number of samples genotyped was smaller than the number of individuals estimated to live in the population for both sampling schemes. Previous studies have shown that genotyping at least twice as many samples as the number of individuals that exist in the study population dramatically decreases the width of the 95% confidence interval surrounding the obtained population estimate [26]-[28].

Despite these limitations, the precision of the genetic estimate is comparable to that of traditional nest count estimates used to evaluate ape population size, for which 95% confidence interval widths (from lower to upper confidence bound) of 40% to 63% of the estimate are reported [14], [15]. Similarly, in our study, using the ML-TIRM model, the total width of the 95% confidence interval surrounding the estimate was 38% of the estimate when applied to the entire four years of data and 61% when using the 2007 data only. Furthermore, with the genetic method, we obtain an absolute minimum number of individuals in the study area and can obtain additional data on minimum group composition and territory size, as well as track individuals over time.

### Recommendations for future ape genetic surveys

We show here that genetic monitoring provides a useful and informative complement to field-based research. Although the 444 chimpanzees samples used here were collected opportunistically over a four-year period, had the focus of a team (or teams) been to search out and obtain faeces, the same number of samples could have been collected over a much shorter period of time. On the other hand, collecting the samples over time allowed us to monitor the movements of individuals. The cost of collection materials and laboratory materials for genetic monitoring are not prohibitive but neither are they trivial. A similar study to the one presented here (695 gorilla samples collected, 384 samples extracted, and 16 microsatellite markers amplified) within the context of a pre-existing field infrastructure, estimated additional laboratory costs for the analysis of the samples to be approximately 12,000 Euros (not including the cost of labor, [23]). Opportunistic sampling can be combined with the regular biomonitoring activities of park rangers, reconnaissance walks, nest decay rate studies by researchers, and/or during the maintenance of remote cameras at field sites, to maximize the use of funds for field activities and research. Without a pre-existing field infrastructure, the incurred costs will be significantly increased, as transport, accommodations, trained field staff, food, etc must all be brought into an area and remain there for an extended period of time as researchers ensure individuals are “recaptured” multiple times. We recommend conducting a pilot study to evaluate sample extraction success, since it can be a major limiting factor as evidenced in this study. Recent advancements in extraction methods should also be attempted if initial extraction success is low [80]. If the sample success rate is extremely low or if samples are difficult to detect and/or chimpanzee density is low, genetic monitoring with opportunistic samples collected over a short period of time may not be feasible. Combining efforts to collect samples for genetic analysis with other new methodologies for detecting elusive species such as scat-detecting dogs [81] and/or video camera trapping [82] should also improve the effectiveness of genetic studies. Further research should focus on validating the genetic capture-recapture method by implementing it under different sampling regimens in an area with a known number of apes. Alternatively, an agent-based model could be used to evaluate the ideal sampling strategy for genetic ape surveys while accounting for the grouping patterns of apes, the variation in habitat types, ape density and sampling area. For example, a recent modeling based approach concluded that orangutan nest surveys can not provide reliable population estimates [18].

For chimpanzees specifically, their fission-fusion social system provides additional challenges to evaluating group composition as samples from individuals in the same social group cannot be linked together as easily as for gorillas [26]; especially if few samples are collected or if extraction success is low. In this study, we used Y-chromosome haplotypes to overcome this challenge, which increases laboratory expenses, but provided us with several interesting insights in the community composition and dynamics at Loango. More extensive study of multiple known chimpanzee communities is needed to examine the assumption that Y-chromosome haplotypes are not shared between groups, while very intensive sampling of unhabituated communities, by showing overlap between the membership of sets of individuals found together, will also serve to build on the analytical foundation presented here. Some aspects of population dynamics such as group extinction (observed here), extra group paternity (as observed in some western chimpanzees,[39], [43]) or moderate territory overlap [36], may make it difficult to attribute some individuals to groups. Most problematic is when males are not identified from all areas, as then many females will go unaffiliated if the recapture rate is low. With better sampling and/or sample success, patterns observed so far only in eastern or western chimpanzee populations could be evaluated for central chimpanzees. For example, if certain males or females are resampled across the entire MCP of their respective groups, this would be inconsistent with the presence of female and/or male neighborhoods in communities of central chimpanzees, at least at Loango.

Genetic surveys can play an important role in assessing wild ape population dynamics when used in addition to traditional surveys, which provide a wealth of information on ape ecology and anthropogenic disturbances. Traditional transect based nest-count surveys can often give rapid assessments to conservation managers that is not possible with genetic-based methods. However, even though genetic surveys will increase the expense of a survey and require increased time for analysis, we demonstrate that the information gained from the additional time and expense is worthwhile, even with opportunistic sampling and a poor success rate. It is clear that opportunistic genetic sampling provides a wealth of information and is a valuable biomonitoring tool for elusive species and we highly recommend its inclusion in forest monitoring activities in the future.

## Supporting Information

Figure S1 Mismatch distributions for the Loango chimpanzee genotypes. The majority of individuals were compared at 8 autosomal loci, however a subset were also compared at 3 additional autosomal loci. Y-chromosome haplotypes were also compared for all the males (with the haplotype coded as a single “homozygous” locus). Values above columns represent number of dyads in each locus category.
(1.10 MB TIF)

Figure S2 Median-joining networks depicting the phylogenetic relationships of Y-chromosomal haplotypes for the Loango chimpanzees. Each circle represents one Y haplotype. Circle size is proportional to haplotype frequency, with the smallest circle representing a haplotype carried by one individual. * denotes haplotypes found in group BI, ^ denotes haplotypes found in group DG.
(2.31 MB TIF)

Figure S3 Movements of individuals C14, C22 and C79 suspected of moving between groups C and A and location of suspected intergroup aggression (infanticides and adult male killing). In June 2006, after following chimpanzee vocalizations, we observed a group of eight chimpanzees that were displaying and vocalizing. Once the chimpanzees had dispersed from the site, bloodspots, chunks of flesh and an infant foot, were found. In June 2007, we observed several chimpanzees vocalizing with hair bristled and appearing distressed. We found fresh blood and bone at the contact site and upon following the group, one male was observed eating what appeared to be an infant chimpanzee. In both cases, diarrhea, a sign of stress, was present at the contact sites. Female C14, originally found in early 2005 with females otherwise associated with group C, was subsequently found in the center of group A's MCP in November 2006. Furthermore, female C22 was found in June 2005 in association with haplotype C male C32 and within the group C MCP in March 2005. She was then found just north (within 300 m) of the group C MCP on three later occasions (April 2006, June 2006, June 2008). In fact, her sample from June 2006 was the only successfully genotyped sample from the nine samples collected in the area of the infant killing described above. In June 2008, C22 was sampled with two males: haplotype C male C79 and haplotype A male C136. Male C79 was initially found in the center of the haplotype C MCP in April 2007 but then in the northeastern limit of group BI's MCP in December 2007 and finally with C136 as described above. Inset: Map of study site (figure 3).
(1.17 MB TIF)

Figure S4 Minimum territory size of group A calculated using male chimpanzee samples with Y-haplotype A that were captured two or more times only. Males sampled more than once noted with their consensus ID. Inset: Map of study site (figure 3).
(1.03 MB TIF)

Table S1 Genotypes of 125 chimpanzees from Loango National Park, Gabon. * individual C12 is an adult male chimpanzee that was killed in the study area in an intercommunity attack in August of 2005 and genotyped in a previous study (Boesch et al 2007).
(0.04 MB DOC)

Table S2 The nine Y-chromosome haplotypes (Y Hap) identified in the Loango Ape Project Study area. The 6 polymorphic loci are shaded in grey with the two alleles of the locus in black or white.
(0.03 MB DOC)

Table S3 Primer sequences, annealing temperature, repeat type and allelic size ranges of Y-chromosomal microsatellite loci. Ta: annealing temperature for singleplex PCR. F: forward primer (the forward primer used in the multiplex and singleplex PCRs are identical except that the forward primer used in the singleplex PCR is fluorescently labeled with FAM, HEX or NED dyes). R: reverse primer. Rnest: reverse nested primer. §: only nested reverse primers were designed for this study, forward and reverse primers are published elsewhere (Erler et al. 2004; Gusmao et al. 2002a; Gusmao et al. 2002b).
(0.02 MB DOC)
