# Supplementary figures and images for: Non-Invasive Genetic Monitoring of Wild Central Chimpanzees
Source: PLoS One. 2011 Mar 15;6(3):e14761. doi: 10.1371/journal.pone.0014761 (PMC3057985; doi:10.1371/journal.pone.0014761)

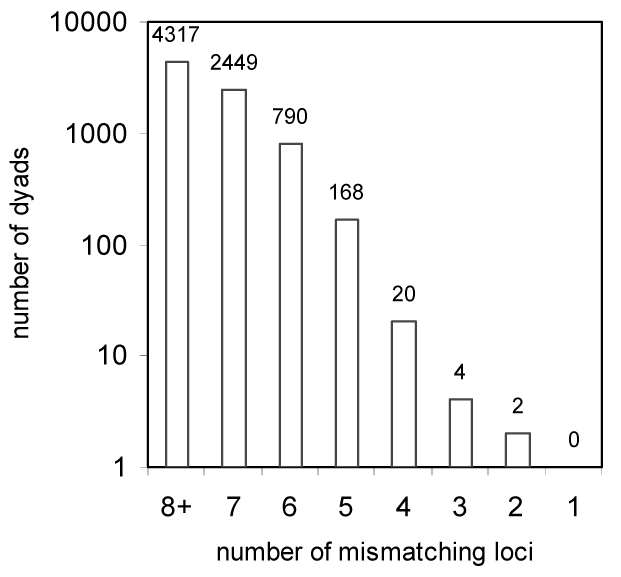

Supplement: Figure S1 — Mismatch distributions for the Loango chimpanzee genotypes. The majority of individuals were compared at 8 autosomal loci, however a subset were also compared at 3 additional autosomal loci. Y-chromosome haplotypes were also compared for all the males (with the haplotype coded as a single “homozygous” locus). Values above columns represent number of dyads in each locus category. (1.10 MB TIF) [file pone.0014761.s001.tif]

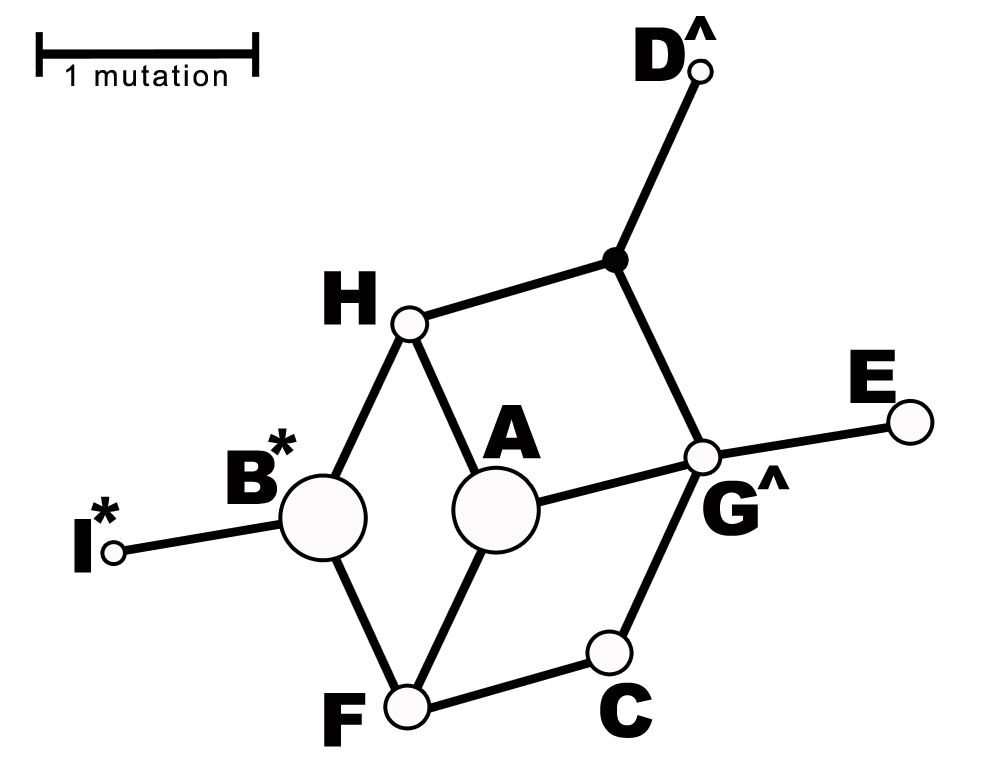

Supplement: Figure S2 — Median-joining networks depicting the phylogenetic relationships of Y-chromosomal haplotypes for the Loango chimpanzees. Each circle represents one Y haplotype. Circle size is proportional to haplotype frequency, with the smallest circle representing a haplotype carried by one individual. * denotes haplotypes found in group BI, ^ denotes haplotypes found in group DG. (2.31 MB TIF) [file pone.0014761.s002.tif]

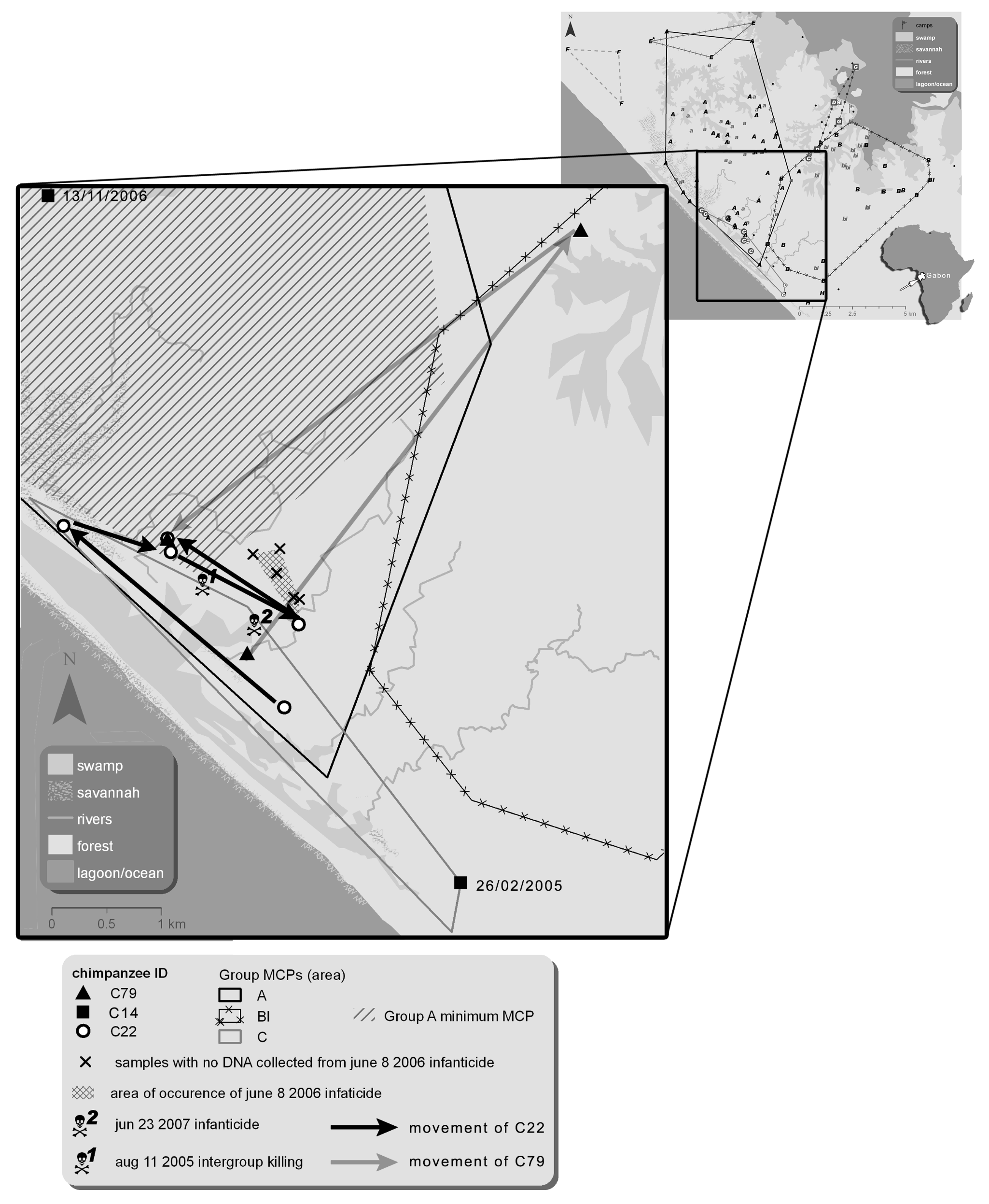

Supplement: Figure S3 — Movements of individuals C14, C22 and C79 suspected of moving between groups C and A and location of suspected intergroup aggression (infanticides and adult male killing). In June 2006, after following chimpanzee vocalizations, we observed a group of eight chimpanzees that were displaying and vocalizing. Once the chimpanzees had dispersed from the site, bloodspots, chunks of flesh and an infant foot, were found. In June 2007, we observed several chimpanzees vocalizing with hair bristled and appearing distressed. We found fresh blood and bone at the contact site and upon following the group, one male was observed eating what appeared to be an infant chimpanzee. In both cases, diarrhea, a sign of stress, was present at the contact sites. Female C14, originally found in early 2005 with females otherwise associated with group C, was subsequently found in the center of group A's MCP in November 2006. Furthermore, female C22 was found in June 2005 in association with haplotype C male C32 and within the group C MCP in March 2005. She was then found just north (within 300 m) of the group C MCP on three later occasions (April 2006, June 2006, June 2008). In fact, her sample from June 2006 was the only successfully genotyped sample from the nine samples collected in the area of the infant killing described above. In June 2008, C22 was sampled with two males: haplotype C male C79 and haplotype A male C136. Male C79 was initially found in the center of the haplotype C MCP in April 2007 but then in the northeastern limit of group BI's MCP in December 2007 and finally with C136 as described above. Inset: Map of study site (figure 3). (1.17 MB TIF) [file pone.0014761.s003.tif]

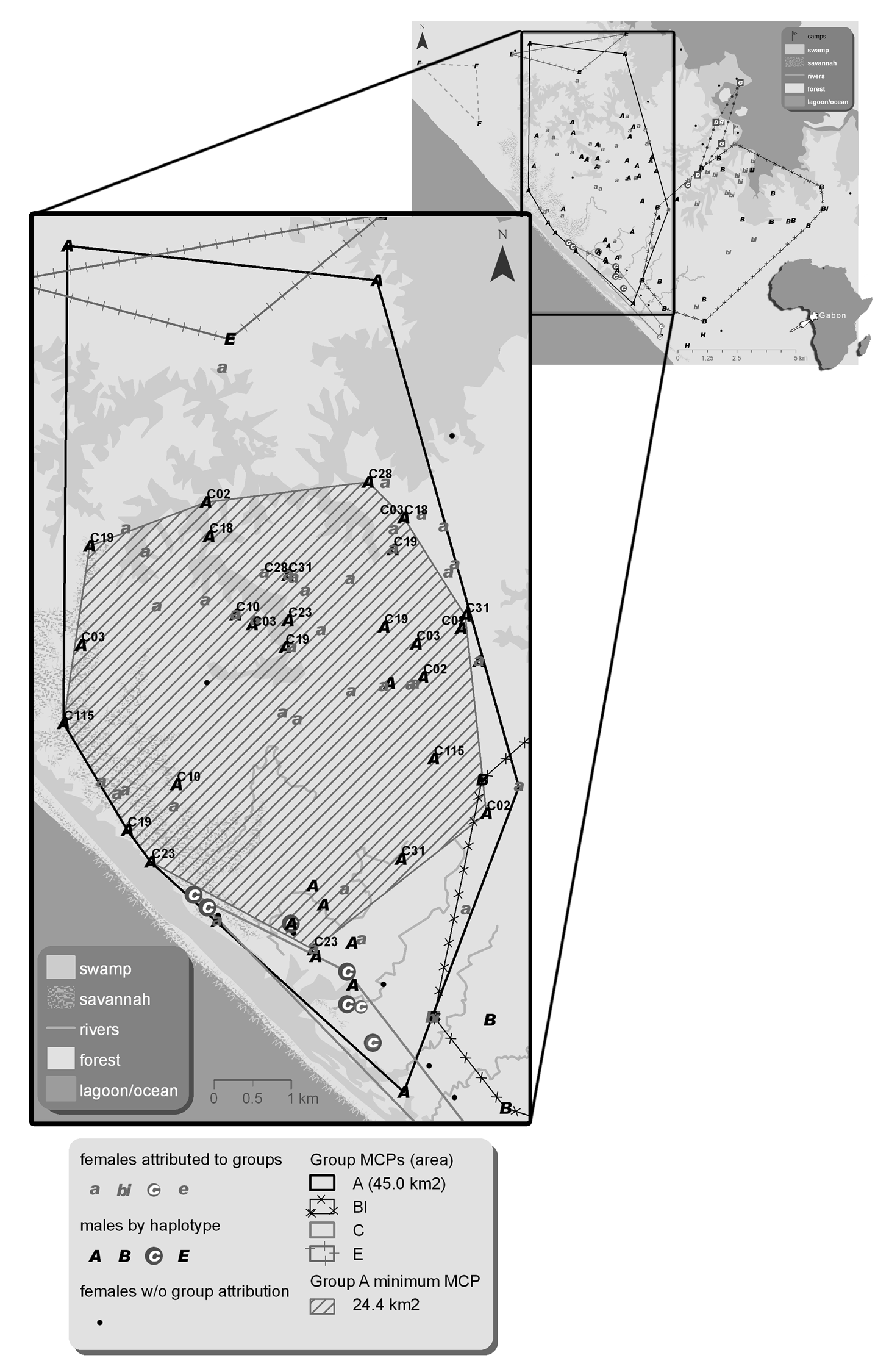

Supplement: Figure S4 — Minimum territory size of group A calculated using male chimpanzee samples with Y-haplotype A that were captured two or more times only. Males sampled more than once noted with their consensus ID. Inset: Map of study site (figure 3). (1.03 MB TIF) [file pone.0014761.s004.tif]
