# Supplementary material for: Non-Invasive Genetic Monitoring of Wild Central Chimpanzees
Source: PLoS One. 2011 Mar 15;6(3):e14761. doi: 10.1371/journal.pone.0014761 (PMC3057985; doi:10.1371/journal.pone.0014761)
Supplement: Supplementary file 2 [file pone.0014761.s005.doc]

consesus	Sex	# of times captured	D3s3038	D5s1470	D6s1056	D9s910	D10s676	D11s2002	D14s306	D16s2624	D1s1622	D1s1656	D4s1627	Y-haplotype
**C01**	M	2	178/198	181/181	234/242	106/118	161/161	141/145	193/229	110/114				A
**C02**	M	5	186/198	181/189	230/234	106/109	161/173	133/137	221/225	110/114				A
**C03**	M	4	182/186	177/185	230/238	103/106	165/169	137/145	221/221	110/110				A
**C04**	F	1	174/182	177/177	234/242	109/115	157/165	145/145	209/221	110/114				
**C05**	F	3	178/190	177/177	238/238	109/115	149/161	129/137	193/213	114/122				
**C06**	M	3	174/182	177/185	230/242	106/112	165/173	141/145	209/237	110/110				A
**C07**	M	1	174/186	177/185	234/254	100/109	165/169	145/145	193/225	114/114				B
**C08**	F	2	182/186	177/185	230/234	109/112	161/165	137/145	217/225	114/122	246/246	122/150	227/231	
**C09**	F	6	178/186	185/185	234/254	100/112	165/165	141/145	193/225	114/114				
**C10**	M	2	182/186	181/189	222/234	106/115	161/165	129/145	197/221	110/130				A
**C11**	F	3	194/194	173/185	238/242	112/115	161/169	145/145	209/213	110/114				
**C13**	F	2	186/186	177/185	226/234	100/109	165/173	141/145	193/205	110/114				
**C14**	F	2	182/194	181/189	234/234	109/115	165/169	137/153	221/229	110/110				
**C15**	F	4	186/186	185/193	230/238	106/106	165/173	129/141	209/209	110/122				
**C18**	M	4	178/198	177/185	242/246	100/112	165/165	145/145	193/197	110/110				A
**C19**	M	5	174/178	173/177	234/242	106/115	165/165	145/145	209/237	110/110	249/249	142/150	211/219	A
**C21**	F	2	190/194	181/189	234/242	109/109	169/173	133/137	221/229	110/110	249/249	142/150	211/219	
**C22**	F	5	182/190	181/189	234/234	112/115	157/169	137/137	233/233	114/114				
**C23**	M	3	174/178	181/193	230/238	106/112	165/169	145/145	193/209	110/110	249/264	134/150	211/231	A
**C24**	F	4	178/178	173/185	234/242	100/109	165/169	145/145	221/225	110/110				
**C25**	M	2	186/186	185/189	226/242	109/112	165/173	129/141	193/221	114/114	246/246	110/122	223/231	B
**C26**	M	2	178/190	177/185	238/242	106/106	161/169	133/153	197/213	122/122				D
**C27**	F	2	178/198	173/177	238/238	103/115	165/169	137/145	213/225	110/110				
**C28**	M	2	174/186	177/185	222/234	115/115	161/161	133/145	221/221	110/126				A
**C29**	F	2	178/186	181/192	238/246	106/112	161/165	145/145	197/225	118/122				
**C30**	F	1	186/186	177/181	222/238	115/115	149/165	141/141	213/233	110/114				
**C31**	M	3	182/186	177/181	238/242	103/106	165/169	137/145	221/221	110/122				A
**C32**	M	3	174/178	173/177	242/242	103/106	165/165	137/145	213/225	110/122				C
**C33**	F	4	174/178	177/192	222/230	106/115	161/165	141/145	221/221	110/126				
**C34**	F	1	186/194	177/181	234/234	109/115	165/169	129/145	201/209	110/110				
**C36**	M	1	178/182	177/181	234/238	103/115	169/169	145/145	221/233	110/114				F
**C37**	M	1	174/178	177/181	230/234	106/115	161/161	129/145	213/221	110/114				F
**C38**	M	1	182/190	173/181	234/234	103/115	161/169	137/145	225/229	114/114				F
**C39**	M	1	186/198	181/189		106/106	165/173	133/137	189/225	110/114				A
**C40**	F	1	182/190	177/181	238/238	112/115	149/165	145/145	209/225	118/126				
**C41**	M	1	174/182	185/189	222/230	100/115	161/161	133/137	221/225	110/122				A
**C42**	F	1	186/190	185/185	222/242	109/115	169/173	137/145	221/233	110/114				
**C43**	F	1	182/186	177/181	238/250	103/115	173/173	129/129	197/221	110/126				
**C44**	F	1	174/186	173/185	226/242	106/115	165/169	145/145	213/221	110/110				
**C45**	M	1	178/182	185/185	238/242	106/106	161/165	129/145	197/209	126/130				A
**C46**	F	1	182/190	181/192	238/238	106/106	157/165	129/149	213/225	110/126				
**C47**	M	1	178/182	181/185	242/242	100/103	161/169	141/145	211/217	110/114				C
**C48**	F	2	178/186	181/185	242/246	103/103	169/169	129/137	221/221	110/110	246/258	138/138	213/225	
**C49**	M	1	174/194	173/181	238/254	100/112	165/165	145/145	221/221	110/114				B
**C50**	M	1	174/174	177/181	238/242	106/112	161/165	129/137	221/225	114/122				B
**C51**	F	1	174/182	177/189	234/254	103/115	165/169	141/145	221/229	114/122				
**C52**	M	1	178/182	177/185	234/234	97/109	161/161	141/145	221/221	110/122				B
**C53**	F	1	178/186	173/177	230/234	103/115	165/165	141/141	209/221	110/126				
**C54**	F	1	174/186	181/185	230/238	109/115	161/173	129/145	213/217	118/126				
**C55**	M	1	182/190	177/177	242/242	115/115	161/173	129/145	211/233	114/126		142/142	215/227	H
**C56**	M	1	174/178	177/177	234/238	115/115	161/165	137/141	197/229	114/122				H
**C58**	M	1	174/178	177/177	234/238	103/115	161/165	137/141	197/229	114/122	249/255	142/162	215/215	B
**C60**	F	2	178/182	173/192	238/238	109/112	161/165	145/145	197/?	118/118	255/?	134/146	215/227	
**C61**	F	1	182/186	177/185	230/234	109/112	161/165	137/145	217/225	122/122	246/270	122/150	227/231	
**C62**	F	1	174/186	173/177	234/254	103/109	161/169	137/145	213/225	110/114				
**C63**	F	1	174/178	181/181	242/246	106/106	169/169	129/141	221/221	110/122				
**C64**	M	1	182/186	173/185	222/242	109/112	161/165	133/145	197/217	110/122				G
**C65**	F	1	182/190	177/185	234/234	103/109	149/161	137/141	221/221	114/122				
**C66**	F	1	178/186	177/192	222/242	103/106	169/173	145/145	221/237	110/110				
**C67**	F	1	178/186	173/177	242/242	103/109	161/165	133/145	197/201	114/122				

consesus	Sex	# of times captured	D3s3038	D5s1470	D6s1056	D9s910	D10s676	D11s2002	D14s306	D16s2624	D1s1622	D1s1656	D4s1627	Y-haplotype
**C68**	M	1	182/182	177/185	238/246	103/118	149/165	141/145	221/229	114/122				A
**C69**	M	1	178/182	177/185	222/238	103/115	165/165	141/145	197/225	106/114				E
**C70**	F	1	174/190	181/181	234/234	106/106	165/169	141/145	197/221	122/122				
**C71**	M	1	178/178	177/177	238/242	106/112	161/173	137/141	197/209	118/122				E
**C72**	F	1	182/186	173/181	246/254	100/106	161/165	145/145	197/209	110/122				
**C73**	M	1	182/182	181/192	222/238	103/?	165/165	141/145	213/229	110/110				B
**C74**	M	1	178/182	181/185	238?/242	106/109	165/165	129/145	193?/197	126/130				A
**C75**	F	1	174/190	173/173	242/242	103/106	161/169	141/141	221/225	110/126				
**C76**	F	1	190/190	185/189	234/238	112/112	161/165	133/145	193/213	114/122				
**C77**	F	1	182/182	185/185	222/250	112/115	149/161	133/145	217/233	110/114				
**C78**	F	1	182/186	185/192	221/246	109/112	149/161	133/141	221/233	110/122				
**C79**	M	3	174/186	185/189	242/242	103/106	169/169	129/145	209/211	110/110				C
**C80**	F	1	174/198	177/185	238/238	103/106	165/169	145/145	221/225	110/110				
**C81**	F	1	182/182	185/189	234/238	106/112	161/161	129/129	197/217	118/130				
**C82**	M	1	186/194	173/177	234/242	103/112	149/161	141/145	221/225	114/114				B
**C83**	F	2	174/178	177/185	222/222	109/112	161/165	133/145	197/221	122/126				
**C84**	M	1	174/182	173/173	238/242	103/112	149/161	141/145	221/225	110/114				B
**C85**	F	1	174/194	173/181	242/246	100/106	161/161	129/145	201/213	110/122				
**C86**	M	1	174/190	177/192	242/254	106/112	149/161	137/141	221/221	110/114				A
**C88**	M	1	174/186	185/189	234/234	109/115	169/169	133/141	221/225	110/114				A
**C89**	F	2	174/198	181/185	230/242	106/115	169/173	141/145	209/225	110/114				
**C90**	F	1	178/182	173/185	242/242	106/109	161/165	137/145	225/225	110/126				
**C91**	F	1	182/190	181/181	230/246	100/109	161/169	137/145	209/209	114/114				
**C92**	F	2	178/178	177/177	230/234	106/115	161/169	141/145	217/221	110/114				
**C93**	M	1	178/186	177/185	222/230	106/115	161/169	133/141	217/221	114/114				A
**C94**	F	1	178/182	177/177	238/238	103/106	149/161	133/145	201/221	110/114				
**C95**	F	1	174/174	181/185	230/254	112/115	165/165	141/145	221/225	110/114				
**C96**	F	1	174/182	185/189	234/234	103/103	149/165	129/145	221/225	114/114				
**C98**	F	1	178/186	173/192	238/246	103/103	161/165	129/141	205/221	110/122				
**C99**	M	1	182/186	173/181	230/234	106/112	161/165	145/145	209/221	110/110				B
**C100**	M	1	182/194	173/177	238/254	103/112	149/169	141/141	221/221	114/114				B
**C101**	F	1	182/186	177/181	238/242	103/115	165/169	133/141	221/221	114/118				
**C102**	M	1	182/198	177/181	234/234	100/115	161/161	137/145	193/225	114/126				B
**C103**	M	3	178/186	173/173	234/246	103/106	153/161	141/145	209/225	110/110				B
**C104**	M	1	174/174	173/177	234/246	103/115	165/165	145/145	221/225	110/130				B
**C105**	M	2	178/182	173/181	242/246	103/115	161/165	145/145	201/225	110/122				B
**C106**	M	2	182/182	177/181	234/242	100/115	161/161	145/145	221/225	114/126				B
**C107**	F	1	178/190	177/181	234/242	109/118	161/169	133/145	197/213	110/122				
**C108**	M	1	174/178	173/185	234/242	103/112	165/169	137/145	225/225	110/114				B
**C109**	M	1	186/190	185/192	234/238	103/112	161/165	145/145	225/225	118/122				B
**C112**	M	2	178/190	181/185	242/246	103/115	149/165	129/141	225/233	110/122				B
**C113**	F	1	182/190	192/192	230/234	100/103	169/173	133/141	221/225	110/114				
**C114**	F	1	182/182	189/192	234/250	100/100	149/169	145/145	201/225	122/122				
**C115**	M	2	182/186	173/181	234/246	106/112	161/169	141/145	217/225	110/114				A
**C116**	M	1	178/194	173/181	234/234	112/115	149/161	129/129	221/233	110/110				F
**C120**	F	1	182/?	181/189	234/238	106/112	161/161	129/145	225/?	110/118				
**C121**	F	1	186/194	185/185	234/238	106/106	161/173	129/141	221/221	110/114				
**C122**	F	1	186/190	189/192	230/238	103/109	161/169	137/145	209/217	110/110				
**C123**	F	1	182/182	173/177	246/254	103/112	161/165	137/145	193/225	114/122				
**C124**	F	1	182/182	173/181	234/234	106/109	153/161	137/145	213/233	110/122				
**C125**	M	1	182/186	173/173	242/246	103/115	161/169	129/129	225/229	100/122				G
**C126**	F	1	178/190	185/185	234/238	106/112	161/169	145/153	197/233	110/122				
**C127**	M	1	182/182	177/177	238/242	115/118	149/161	133/145	221/225	118/118				A
**C128**	F	1	182/186	181/181	230/246	102/115	165/165	129/145	217/221	110/114				
**C129**	M	1	182/182	181/185	238/242	102/115	157/165	145/145	197/211	114/122				E
**C130**	M	1	174/198	173/181	230/230	106/112	165/169	145/145	209/?	110/122				A
**C131**	F	1	186/186	173/177	234/234	115/115	169/169	133/137	233/?	118/122				
**C133**	F	1	182/186	181/181	234/234	103/103	161/169	133/145	209/213	110/110				
**C134**	M	1	178/186	181/185	234/238	109/109	165/165	145/145	193/225	122/122				I
**C135**	F	1	174/190	189/189	242/242	100/112	161/161	133/141	193/205	122/130				
**C136**	M	1	182/190	189/189	234/234	112/112	169/169	137/145	193/233	114/114				A
**C12***	M	1	178/186	173/185	222/222	106/115	161/169	137/145	221/233	114/122				C
