# Supplementary material for: Non-Invasive Genetic Monitoring of Wild Central Chimpanzees
Source: PLoS One. 2011 Mar 15;6(3):e14761. doi: 10.1371/journal.pone.0014761 (PMC3057985; doi:10.1371/journal.pone.0014761)
Supplement: Supplementary file 3 [file pone.0014761.s006.doc]

	Y-chromosome loci												
**Y Hap**	**DyS612**	**DyS562**	**DyS510**	**DyS588**	**DyS533**	**DyS517**	DyS469	DyS632	DyS502	DyS439	DyS520	DyS630	DyS392
**A**	**185**	**178**	**175**	**154**	**190**	**193**	182	137	190	217	144	154	168
**B**	**188**	**174**	**175**	**154**	**190**	**193**	182	137	190	217	144	154	168
**C**	**185**	**174**	**175**	**154**	**186**	**193**	182	137	190	217	144	154	168
**D**	**188**	**178**	**175**	**159**	**186**	**193**	182	137	190	217	144	154	168
**E**	**185**	**178**	**175**	**154**	**186**	**197**	182	137	190	217	144	154	168
**F**	**185**	**174**	**175**	**154**	**190**	**193**	182	137	190	217	144	154	168
**G**	**185**	**178**	**175**	**154**	**186**	**193**	182	137	190	217	144	154	168
**H**	**188**	**178**	**175**	**154**	**190**	**193**	182	137	190	217	144	154	168
**I**	**188**	**174**	**179**	**154**	**190**	**193**	182	137	190	217	144	154	168
