# Supplementary material for: Non-Invasive Genetic Monitoring of Wild Central Chimpanzees
Source: PLoS One. 2011 Mar 15;6(3):e14761. doi: 10.1371/journal.pone.0014761 (PMC3057985; doi:10.1371/journal.pone.0014761)
Supplement: Supplementary file 4 [file pone.0014761.s007.doc]

Locus	T_a (°C)	Repeat type		Primer Sequences (5’ – 3’)[§]
DYS392	56	tri	F:	TCATTAATCTAGCTTTTAAAAACAA
			R:	AGACCCAGTTGATGCAATGT
			R_{nest}:	CAGAGGGATCATTAAACCTACC
DYS439	59	tetra	F:	TCCTGAATGGTACTTCCTAGGTTT
			R:	GCCTGGCTTGGAATTCTTTT
			R_{nest}:	GAATTCTTTTACCCATCATCTCTTTAC
DYS469	59	tri	F:	TTTGGGGACTGAATTCAAAA
			R:	CCCCAGCTGGTAAAATGAGT
			R_{nest}:	TTTCTCTCAGAACAATGGCAGT
DYS502	59	tri	F:	CAGCAAGCCACCATACCATA
			R:	TGTGCTTTTGGAGTTTGGAG
			R_{nest}:	TCACTGTGATGAACCCTGATG
DYS510	58	tetra	F:	TTTTTCCTCCCTTACCACAGA
			R:	TCTGGAGAAGACAGAACTTGTCA
			R_{nest}:	TCTGCCTAATCATCCATCCA
DYS517	58	tetra	F:	TAATCGTCCCATTTTGAGCA
			R:	TGCAATCCCAAACTCAGAAA
			R_{nest}:	CAGAAATTCATTCAAGTTGTTTCC
DYS520	60	tetra	F:	AACAGCCTGCCCAACATAGT
			R:	ACCATCATGCCCTGCAATA
			R_{nest}:	CATCATGCCCTGCAATATTG
DYS533	55	tetra	F:	CATCTAACATCTTTGTCATCTACC
			R:	TGATCAGTTCTTAACTCAACCA
			R_{nest}:	GTTCTTAACTCAACCAAACAATAG
DYS562	62	tetra	F:	GGGTGTATAAAGAGGGGCATA
			R:	GGTAAAGGTTATCACGCCATC
			R_{nest}:	GGTAAAGGTTATCACGCCATC
DYS588	61	penta	F:	GAATGCAGAACCCTCAAGGA
			R:	AGCCTGGGTGACAGAAACAC
			R_{nest}:	CCTGGGTGACAGAAACACATTC
DYS612	62	tri	F:	CCCCCATGCCAGTAAGAATA
			R:	TGAGGGAAGGCAAAAGAAAA
			R_{nest}:	TGAGGGAAGGCAAAAGAAAATA
DYS630	59	tetra	F:	GCCTTTGGACAGAGCAAGAC
			R:	AGCCATGGAAAGCTGTGAGT
			R_{nest}:	CATGGAAAGCTGTGAGTTCC
DYS632	61	tetra	F:	GGCCGTTGCAAAATAAACTG
			R:	TCTGGGCAACAGAAGGAGAC
			R_{nest}:	GCAACAGAAGGAGACTCCATCTC
